# Genome-Wide Analysis of the *JAZ* Family in *Brassica rapa* and the Roles of *BrJAZ1a* and *6b* in Response to Stresses

**DOI:** 10.3390/ijms27010289

**Published:** 2025-12-27

**Authors:** Chuang Liang, Qingchang Feng, Xingliang Wang, Kaixin Li, Zhixu Li, Yan Zhang, Yaowei Zhang, Yan Liu

**Affiliations:** 1 College of Horticulture and Landscape Architecture, Northeast Agricultural University, Harbin 150030, China; 2 Key Laboratory of Biology and Genetic Improvement of Horticulture Crops (Northeast Region), Ministry of Agriculture and Rural Affairs, Northeast Agricultural University, Harbin 150030, China; 3 Horticultural Branch, Heilongjiang Academy of Agricultural Sciences, Harbin 150060, China

**Keywords:** Chinese cabbage, *JAZ*, gene family, abiotic stress

## Abstract

Jasmonate-ZIM domain (JAZ) proteins act as repressors in the jasmonic acid (JA) signaling pathway and also function as plant-specific proteins participating in plant growth and development, stress response, and defense. In our study, a total of 25 *JAZ* genes were identified in *B. rapa* based on their conserved domains. First, the primary characteristics were surveyed, including the lengths of the CDS and proteins, molecular weights, and isoelectric points. Next, a phylogenetic tree of JAZ proteins among *B. rapa*, *A. thaliana*, *O. sativa*, *B. oleracea*, and *B. napus* was constructed, which revealed that these proteins cluster into four groups based on sequence homology rather than by species. Synteny analysis of *JAZ* genes among these species demonstrated that the highest number of collinear pairs was found between *B. rapa* and *B. napus*. Most *BrJAZ* genes were highly expressed in root, stem, and leaf. Moreover, the expression levels of *BrJAZ1a* and *BrJAZ6b* were induced by drought, high salt, black rot, and MeJA. Over-expressed these genes in *A. thaliana* lines enhanced their tolerance to drought and high salt stress, which was associated with higher enzymatic activities of SOD and POD. Both *BrJAZ1a*-GFP and *BrJAZ6b*-GFP were localized in the nucleus.

## 1. Introduction

The *JAZ* gene family represents a subgroup of the TIFY transcription factors and is defined by two highly conserved domains: the ZIM domain, which facilitates protein–protein interactions, and the Jas domain, which plays a critical role in jasmonic acid (JA) signal transduction [1]. JAZ proteins act as central repressors in the JA signaling pathway by interacting with transcription factors such as MYC, thereby modulating the expression of downstream genes [2]. Through this regulatory mechanism, JAZ proteins exert significant influence on plant growth, development, and responses to environmental stimuli.

JA signaling also works in combination with multiple hormones in plant responses to abiotic stresses, such as drought, salinity, and low temperature [3]. Accumulating evidence across various plant species demonstrates that JAZ proteins contribute to stress adaptations by integrating with particularly abscisic acid (ABA). For instance, *JAZ1* has been reported to act as a positive regulator in seed germination in bread wheat by directly interacted with *TaABI5*, leading the repression of the ABA pathways [4]. Furthermore, *JAZ1*, *JAZ6*, and *JAZ9* were rapidly induced by drought and salt stress in *Oryza sativa* [5]; moreover, *OsJAZ9* enhanced its tolerance to potassium deficiency, while its knockdown lines were sensitive [6]. On the other hand, *JAZ1* was induced at 3 h of drought stress in sugarcane. Furthermore, its over-expressed transgenic *Arabidopsis thaliana* lines promoted the flowering time to be shorter than Col-0 [7]. *JAZ25* acts as a negative regulator in gray leaf spot resistance by directly binding on the SlMYC2 promoter in *Solanum lycopersicum* [8]. In cruciferous plants, there were not only the 13 *JAZ* family members identified in *A. thaliana* in response to biotic and abiotic stress responses [9], but also the genome-wide identification and functional analyses of *JAZ* members in turnip (*Brassica rapa*), *Brassica napus*, have been carried out [10,11,12]. These studies in *A. thaliana* showed that *JAZ8* acts as a negative regulator against *Botrytis cinerea* infection [13], and *JAZ7* promoted drought tolerance [14], while *JAZ1* and *JAZ4* play negative roles in freezing stress [15].

*B. rapa* is famous for its high nutritional value and low price with widespread cultivation in East Asia and suffers various abiotic and biotic stresses in field, such as drought, salinity, low temperature, and bacterial diseases, leading losses of crop yield and quality [16]. It is reported that *JAZ* genes play roles in both abiotic and biotic stresses; however, there were scarcely any reports of the *JAZ* family in *B. rapa.* In our study, 25 *JAZ* genes of Chinese cabbage were identified and analyzed, such as chromosomal locations, promoter cis-elements, the protein motifs, gene structures, molecular weights, and isoelectric points. To investigate the evolutionary relationships of the *JAZ* members among multiple species, a phylogenetic tree of JAZ proteins was built among *B. rapa*, *A. thaliana*, *O. sativa*, *B. oleracea*, and *B. napus*. Furthermore, synteny analysis of *JAZ* genes among *B. rapa* and the other species was conducted to demonstrate their duplication events. The expression levels of *BrJAZs* in different tissues and various treatments, including drought, salinity, black rot infection, and MeJA, were further detected by qPCR. To identify the function of *BrJAZ* in *B. rapa*, *BrJAZ1a* and *BrJAZ6b* were selected and overexpressed in *A. thaliana* for drought and high salt treatments.

## 2. Results

### 2.1. The Isolation and Identification of the JAZ Family Genes in B. rapa

1. A total of 31 candidate *JAZ* family members were initially identified in *B. rapa* via annotation and homology searches in the BRAD database. The presence of the characteristic ZIM domain in these candidate proteins was then validated using the InterPro (http://www.ebi.ac.uk/interpro/, accessed on 20 February 2025), SMART (http://smart.embl-heidelberg.de/, accessed on 20 February 2025), and ScanProsite (https://prosite.expasy.org/, accessed on 20 February 2025) tools. This analysis confirmed that only 25 genes contained a typical TIFY domain, and these were retained for further study. Based on their homology to *A. thaliana JAZ* genes, these 25 genes were designated as *BrJAZ1* to *BrJAZ12* (Table S1). Notably, no homologous genes for *AtJAZ11* and *AtJAZ12* were found in the Chinese cabbage genome. The key characteristics of the 25 *BrJAZ* genes are summarized in Table 1. The CDS lengths ranged from 342 bp to 1071 bp, while the genomic sequences were considerably longer (501 bp to 3273 bp), a difference attributable to the variation in intron number and size of the introns. The encoded proteins ranged from 113 to 356 amino acids in length, with molecular weights between approximately 13.0 kDa and 39.8 kDa. The theoretical isoelectric points (pI) of most BrJAZ proteins were basic, ranging from 7.9 to 10.07, except for *BrJAZ12a*, which had an acidic pI of 4.97.

### 2.2. Phylogenetic Relationships and Synteny of JAZ Members Among B. rapa, A. thaliana, B. napus, and B. oleracea

**Figure 1:**
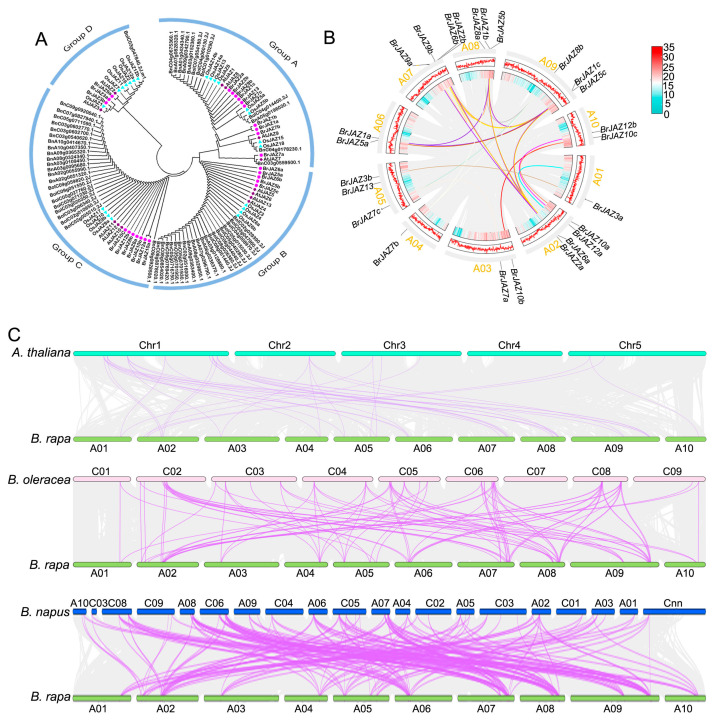
Phylogenetic and collinearity analysis of *JAZ* family members. (**A**) Phylogenetic relationships of JAZ proteins among *B. rapa*, *A. thaliana*, *B. oleracea*, and *B. napus*. (**B**) Collinearity analysis of *BrJAZ* genes within the *B. rapa* genome. (**C**) Synteny analysis of *JAZ* genes between *B. rapa* and three related species (*A. thaliana*, *B. oleracea*, and *B. napus*).

Firstly, the phylogenetic analysis of 121 JAZ proteins derived from *B. rapa*, *A. thaliana*, *B. napus*, *B. oleracea*, and *O. sativa* was conducted by the maximum likelihood method including 13 *AtJAZs*, 23 *OsJAZs*, 25 *BraJAZs*, 21 *BolJAZs*, and 38 *BnJAZs*. These proteins were classified into four major subgroups, designated Group A to D (Figure 1A). Group C was the largest with 37 members, which were clustered by species rather than orthology. In contrast, Group D is the smallest, including 13 members and clustering based on gene homology, such as *JAZ* members of *B. rapa* and *A. thaliana* grouping together.

Collinearity analysis of the *BrJAZ* genes revealed 33 collinear pairs (Figure 1B) listed in Table S2. Furthermore, synteny analysis of *JAZ* members between *B. rapa* and *A. thaliana*, *O. sativa*, *B. oleracea*, and *B. napus* was performed (Figure 1C, Table S3). Numerous collinear pairs were detected between *B. rapa* and other cruciferous species: 39 pairs with *A. thaliana*, 91 pairs with *B. oleracea*, and 154 pairs with *B. napus* (Table S3). In contrast, no collinear pairs were identified between *B. rapa* and *O. sativa* (Figure S1).

### 2.3. The Gene Localization, Structures, and Conserved Motifs of BrJAZ Members

**Figure 2:**
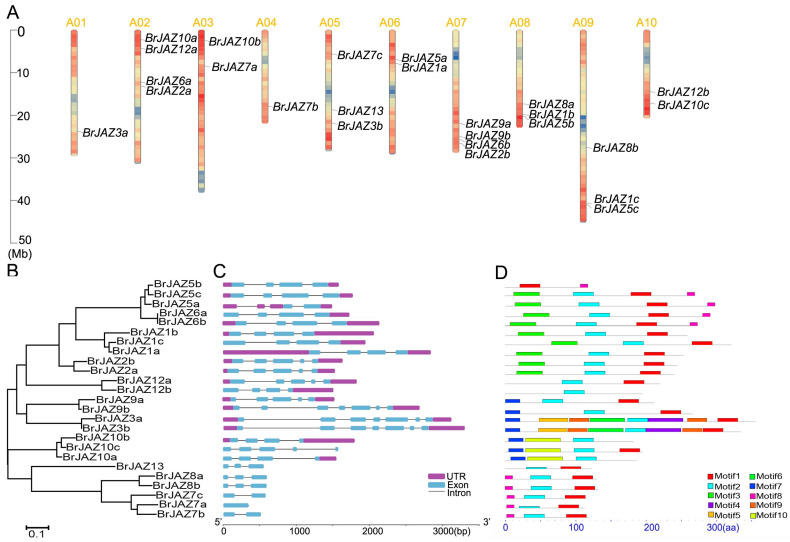
Chromosomal localization, phylogenetic tree, exon–intron structure, and conserved motifs of *BrJAZ* genes. (**A**) Chromosomal distribution of *BrJAZ* genes in *B. rapa*. (**B**) Phylogenetic tree of BrJAZ proteins. (**C**) Exon–intron structure of *BrJAZ* genes. (**D**) Conserved motifs in BrJAZ proteins.

The 25 *BrJAZ* genes were distributed unevenly across all ten chromosomes of *B. rapa*. Chromosomes A02 and A07 each contained four genes, whereas only one gene each was located on A01 (*BrJAZ3a*) and A04 (*BrJAZ7b*). Three chromosomes (A05, A08, and A09) harbored three *BrJAZ* genes each, and the remaining chromosomes each contained two members (Figure 2A). These results suggested the occurrence of both tandem and segmental duplications in the *BrJAZ* gene family. A phylogenetic tree of the 25 BrJAZ proteins was constructed using the Neighbor-Joining method, clustering them into three major groups (Figure 2B). In addition, ten conserved motifs were identified in the BrJAZ proteins (Figure 2D) with the sequence logos presented in Figure S2. The most conserved motif, Motif 2, presented in 24 members, following by Motif 1 in 21 proteins (Figure 2D). The exon–intron structures of the *BrJAZ* genes are shown in Figure 2C. Nearly half of the genes contain four exons, and members within the same phylogenetic subgroup exhibit similar gene structures.

### 2.4. The Cis-Acting Elements on the BrJAZ Promoters

**Figure 3:**
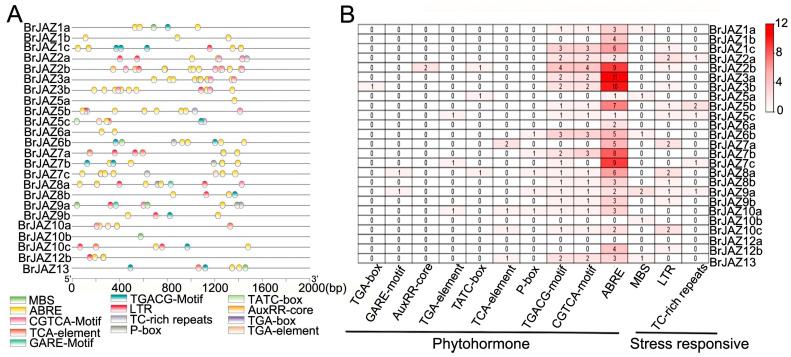
Cis-acting elements related to abiotic stress in *BrJAZ* promoters. (**A**) Distribution of cis-acting elements related to abiotic stress. (**B**) Number of each cis-acting element.

The 2000 bp promoter sequences upstream of the start codon of each *BrJAZ* gene were retrieved for cis-element analysis (Figure 3). Hormone-responsive elements were identified in most *BrJAZ* promoters, except for *BrJAZ10b* and *BrJAZ12a*. Notably, the MeJA-responsive motifs TGACG and CGTCA were present in 18 *BrJAZ* promoters, suggesting these genes are involved in the JA signaling pathway. The most abundant elements were ABREs, associated with abscisic acid responsiveness, with 109 copies distributed across 23 *BrJAZ* promoters. several stress-related cis-elements were detected, including MBS (drought stress), LTR (low-temperature stress), and TC-rich repeats (general stress response) (Figure 3A,B). These results revealed that *BrJAZ* genes are involved in hormone responses and stress responses.

### 2.5. The Expression Levels of BrJAZs in Different Tissues and in Response to Stresses

**Figure 4:**
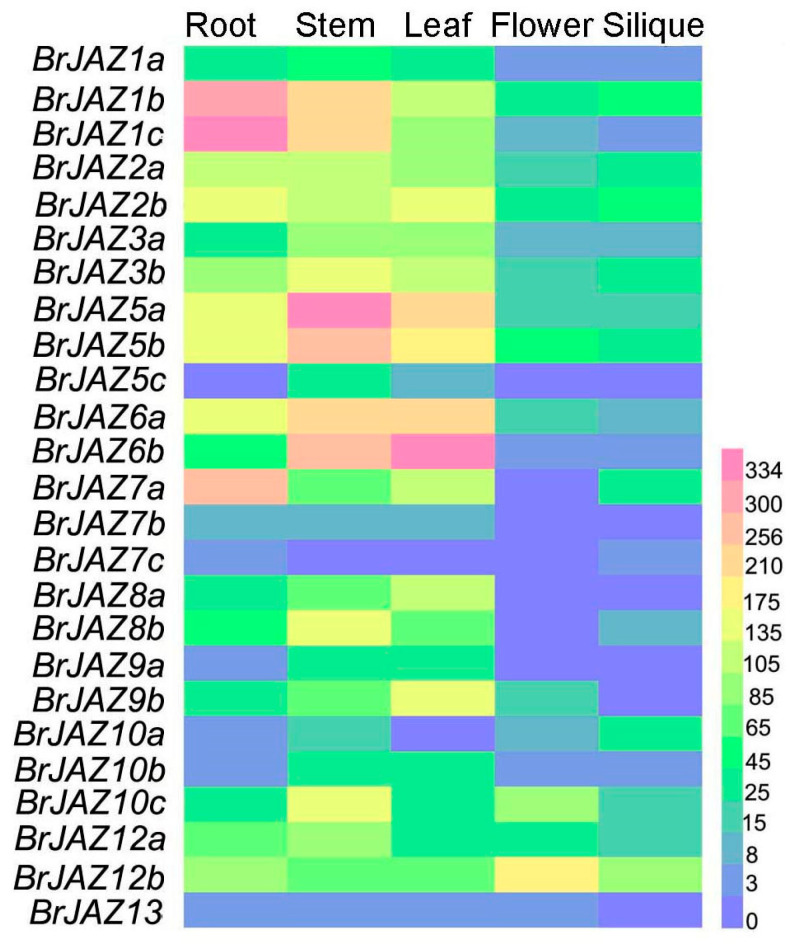
The expression patterns of *BrJAZ* members in in root, stem, leaf, flower, and silique.

The expression levels of *BrJAZ* genes in different tissues (root, stem, leaf, flower, and silique) were analyzed using transcriptome data of Chinese cabbage from the BRAD database. Most *BrJAZ* genes exhibited relatively high expressions in root, stem, and leaf (Figure 4). *BrJAZ5a*, *BrJAZ5b*, *BrJAZ6a*, and *BrJAZ6b* were markedly induced in root and leaf, whereas *BrJAZ1b* and *BrJAZ1c* showed high transcript levels in root, stem, and leaf. In contrast, the expression levels of *BrJAZ7b*, *BrJAZ7c*, and *BrJAZ13* were low across each tissue (Figure 4).

### 2.6. The Transcriptional Levels of BrJAZs in Response to Stresses

**Figure 5:**
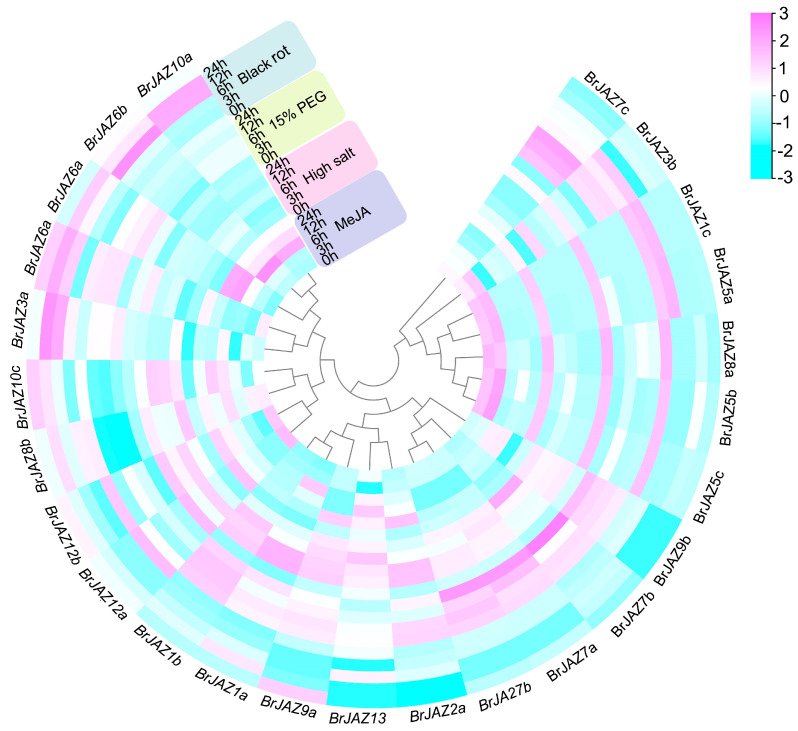
qPCR analysis of *BrJAZ* gene expression in response to drought, salt stress, black rot infection, and MeJA treatment at 0, 3, 6, 12, and 24 h.

The transcriptional levels of *BrJAZ* genes in *B. rapa* under drought and high-salt stresses were further detected by qPCR at 0, 3, 6, 12, and 24 hours post-treatment (Figure 5). Under drought stress, nine members (*BrJAZ1a*, *BrJAZ1b*, *BrJAZ2a*, *BrJAZ2b*, *BrJAZ6a*, *BrJAZ7a*, *BrJAZ7b*, *BrJAZ9a*, and *BrJAZ9b*) were markedly induced, with peak expression levels generally observed at 3 h and 6 h. Among them, *BrJAZ1a*, *BrJAZ1b*, *BrJAZ7a*, and *BrJAZ7b* maintained notably high expression from 3 h to 24 h (Figure 5). Under high-salt stress, *BrJAZ1a*, *BrJAZ1b*, *BrJAZ2a*, and *BrJAZ9a* were strongly induced at 24 h, whereas *BrJAZ2b* was up-regulated at the earlier time points of 3 h and 6 h (Figure 5). Furthermore, the expression of *BrJAZ1a*, *BrJAZ6a*, *BrJAZ6b*, *BrJAZ10a*, and *BrJAZ10b* was significantly enhanced by both MeJA treatment and black rot pathogen infection. In contrast, *BrJAZ2a* and *BrJAZ9a* were induced specifically by MeJA at 24 h, and *BrJAZ3a* was specifically up-regulated in response to black rot infection (Figure 5).

### 2.7. The Phenotypic Characteristics of Over-Expressed BrJAZ1a and BrJAZ6b Transgenic A. thaliana Lines Under Abiotic Stresses

**Figure 6:**
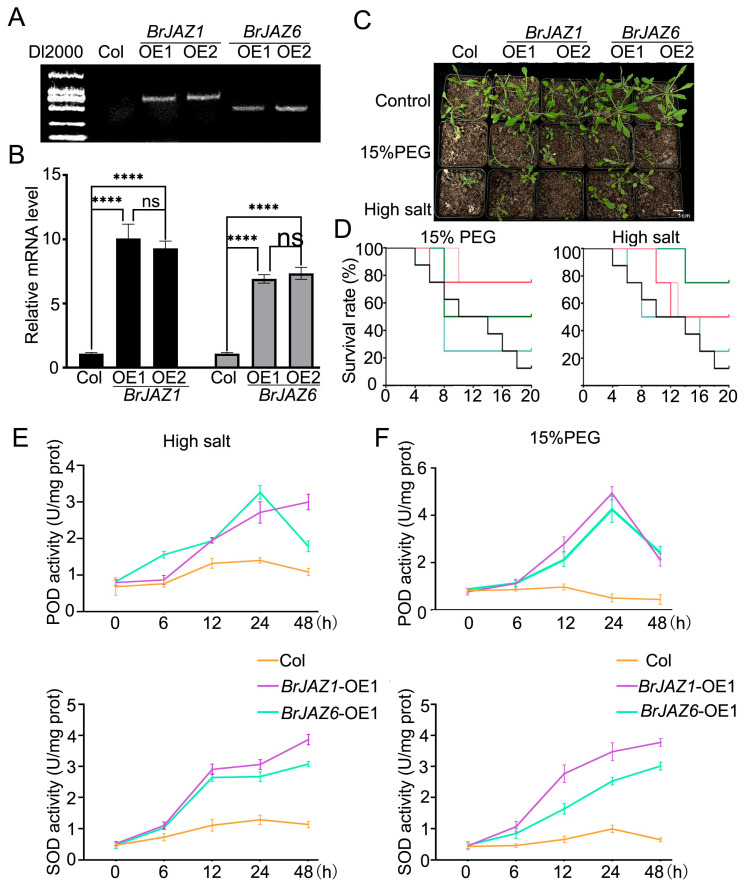
Phenotypic and physiological analyses of *BrJAZ1a*- and *BrJAZ6b*-overexpressing *A. thaliana* lines under abiotic stress. (**A**) PCR confirmation of transgenic *BrJAZ1a* and *BrJAZ6b* overexpression lines. (**B**) Expression levels of *BrJAZ1a* and *BrJAZ6b* in transgenic lines by qPCR. (**C**) Phenotype of wild-type (Col-0) and transgenic lines under drought (15% PEG) and salt (200 mM NaCl) stress. (**D**) Survival rates of the indicated lines under drought and salt stress over time. (**E**) POD and SOD activities in wild-type and transgenic lines under salt stress. (**F**) POD and SOD activities in wild-type and transgenic lines under drought stress. Data are presented as mean ± SD of three biological replicates. Statistical significance was determined by two-way ANOVA followed by multiple comparisons tests (**** *p* < 0.0001; ns, not significant).

*BrJAZ1a* and *BrJAZ6b* were over-expressed in *A. thaliana* Col-0 by *Agrobacterium tumefaciens*-mediated transformation. Two independent transgenic lines for each gene were obtained through hygromycin selection and confirmed by PCR using gene-specific primers (Figure 6A; Table S4). The expression levels of *BrJAZ1a* in the OE1 and OE2 lines were up to 10.077- and 9.293-fold higher than in Col-0, respectively, while *BrJAZ6b* expression was up to 6.927- and 7.350-fold higher (Figure 6B). The transgenic lines and Col-0 were subjected to drought and high-salt treatments. The survival rates of the transgenic lines were higher than Col-0. The activities of superoxide dismutase (SOD) and peroxidase (POD) were measured in these lines under drought and salt stress at 0, 6, 12, 24, and 48 h (Figure 6D,E). Under drought stress, POD activities of the *BrJAZ1a* and *BrJAZ6b* overexpression lines were induced, peaked at 24 h and then declined at 48 h, remaining higher than in Col-0. Under salt stress, POD activities of the *BrJAZ6b* lines showed a similar trend, whereas in the *BrJAZ1a* lines, they increased continuously throughout the treatment. SOD activities increased continuously following both stress treatments; moreover, these of the overexpression lines had significantly higher activity than Col-0 (Figure 6D,E).

### 2.8. The Subcellular Localization of BrJAZ1a-GFP and BrJAZ6b-GFP

**Figure 7:**
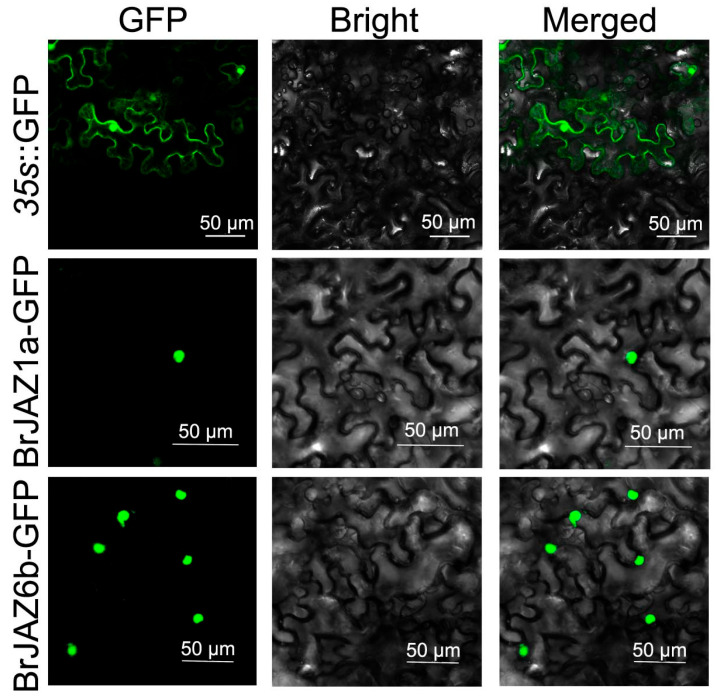
The subcellular localization of *BrJAZ1a*-GFP and *BrJAZ6b*-GFP in *N. benthamiana*.

To determine the subcellular localization of *BrJAZ1a* and *BrJAZ6b*, *BrJAZ1a*-GFP and *BrJAZ6b*-GFP fusion constructs were generated and transiently expressed in *N. benthamiana* leaves. Fluorescence signals were observed using confocal microscopy. The *35s*::GFP exhibited the green fluorescence in the nucleus, cytoplasm, and membrane. In contrast, both *BrJAZ1a*-GFP and BrJAZ6b-GFP were specifically localized in the nucleus, as shown in Figure 7.

## 3. Discussion

Jasmonate ZIM-domain (JAZ) proteins are central repressors of JA signaling and play critical roles in plant growth, development, and stress responses. Increasing evidence indicates that JAZ-mediated regulations are pivotal for plant adaptation to both abiotic and biotic stresses, including drought, salinity, and pathogen infection. *B. rapa*, an important leafy vegetable crop, is frequently exposed to multiple environmental stresses throughout its growth cycle, which significantly constrain yield and quality [17]. Extensive studies of *JAZ* genes in *Arabidopsis* and other *Brassica* species were identified; however, a systematic investigation of the *JAZ* gene family in *B. rapa* has been lacking. In this study, 25 *JAZ* family members were identified in *B. rapa* with the main information listed in Table 1, including the length of CDS and protein, exon number, molecular weight, and isoelectric point. Next the protein phylogenetic tree and motifs of BrJAZs exhibit that the motifs of the same clustered groups were similar. Furthermore Motif 1 (ZIM domain) and Motif 2 (Jas domain) are the most conserved, presenting in nearly every member, while other motifs vary in number and arrangements (Figure 2D). Similar conserved motifs of the *JAZ* families have been reported in *Arabidopsis*, *B. oleracea*, and *B. napu* [12,18,19,20], indicating that core *JAZ* functions are evolutionarily conserved across Brassicaceae species. It has been reported that the *JAZ* family has 13 members in *A. thaliana* [18], while there were 15 members in rice [20] and 26 and 56 members in *B. oleracea* [12] and *B. napus* [19], respectively, suggesting that gene duplication has driven the expansion and diversification of the *JAZ* family in Chinese cabbage. Therefore, we built the evolutionary relationships of *JAZ* members among these species, they were clustered together based on gene homology rather than species-specific relationships, indicating that the *JAZ* genes are conserved in different species. Synteny revealed abundant collinearity pairs of *JAZ* genes between *B. rapa* and other species but none were found in rice, reflecting the evolutionary divergence between dicots and monocots.

The promoters of most *BrJAZ* genes harbored multiple hormone- and stress-related elements, including ABRE (ABA-responsive), MBS (drought-responsive), and CGTCA/TGACG motifs (MeJA-responsive). Notably, ABRE elements—known mediators of ABA-dependent stress responses [21]—were present in nearly all *BrJAZ* promoters, implying potential crosstalk between ABA and JA signaling. The widespread occurrence of MeJA-responsive elements further supports the role of *BrJAZs* in JA-mediated defense, which is consistent with reports in other Brassica species [10,22,23].

The expression levels of *BrJAZ* genes were further monitored in various responses including drought, high salinity, MeJA treatment, and black rot infection. Ten *BrJAZ* genes were significantly induced by drought stress, while nine members were increased under high salinity. The expressions of six *BrJAZ*s were markedly induced by exogenous MeJA, while *BrJAZ1a/1b*, *BrJAZ3a*, *BrJAZ6a/6b*, and *BrJAZ10* were significantly upregulated by black rot stresses. It has been reported that the expression levels of *JAZ* genes were up-regulated under multiple stresses in turnip, rapeseed, and *Brassica juncea* [10,23,24], which aligns with our study; together with the results in our study, this suggests a conserved mechanism. Notably, *BrJAZ1a* and *BrJAZ6b* were strongly induced in each treatment, indicated that *BrJAZ1a* and *BrJAZ6b* might play central roles in the broad stress adaptation in *B. rapa*. Therefore, *BrJAZ1a* and *BrJAZ6b* were highly over-expressed in *Arabidopsis*, which showed that they enhanced the drought and salt tolerance of their over-expressed transgenic lines compared to Col-0, with the increased activities of antioxidant enzymes (e.g., SOD and POD). It has been reported that *JAZ1* and *JAZ6* play roles in the stress responses of many species, such as *TaJAZ1* promoting the seed germination in bread wheat [4]; *OsJAZ1* and *OsJAZ6* were rapidly induced by drought and salt stress in *O. sativa* [5]; and overexpressing *GaJAZ1* increased tolerance to salt stress in *G. hirsutum* [25]. Furthermore research findings on the mechanism of JAZ’s involvement in abiotic stress as core repressors of jasmonic acid (JA) signaling revealed that jasmonate ZIM-domain (JAZ) proteins directly interact with MYC transcription factors to modulate JA-responsive gene expression [26,27] Under normal conditions, JAZ proteins inhibit MYC activity; however, stress-induced JA accumulation triggers JAZ degradation via the SCFCOI1 complex, thereby releasing MYC to activate defense pathways in *Arabidopsis* [28,29]. Notably, specific *JAZ* members are known to fine-tune—rather than simply repress—JA signaling, enabling an adaptive responses to abiotic stress [30]. Our study provides that *BrJAZ1a* and *BrJAZ6b* are located in the nucleus and play positive roles in drought and salt stresses in *Arabidopsis*; however, the molecular mechanisms were not explored. Collectively, our findings offer valuable genetic resources and theoretical framework for breeding stress-resilient Brassica crops.

### Limitations and Future Perspectives

This study provides a comprehensive genomic and functional characterization of the *JAZ* gene family in *B. rapa*, identifying *BrJAZ1a* and *BrJAZ6b* as positive regulators of drought and salt tolerance. However, since the genetic transformation system of Chinese cabbage has not obtained transgenic plants in *B. rapa* and only subcellular localization experiments were conducted, definitive conclusion cannot be drawn without further exploring how *BrJAZ1a* and *BrJAZ6b* are involved in non-biological molecular mechanisms. In future studies, the functions and underlying mechanisms of these two genes will be investigated in depth.

## 4. Materials and Methods

### 4.1. Plant Materials

Seeds of the Chinese cabbage inbred line and *A. thaliana* ecotype Col-0 were obtained from the Chinese Cabbage Research Laboratory, College of Horticulture and Landscape Architecture, Northeast Agricultural University. Chinese cabbage plants were cultivated in a greenhouse under a 16-h light (light intensity: 200 µmol m^−2^ s^−1^, temperature: 25 °C)/8-h dark (temperature: 20 °C) photoperiod. The relative humidity was controlled at 60–70%, and the growth substrate was a mixed medium of peat moss:vermiculite:perlite at a volume ratio of 3:1:1. Upon reaching the five-leaf stage, some of the plants were subjected to treatment with 100 µM ABA (foliar spray), 100 µM MeJA (foliar spray), 15% PEG 8000 (root irrigation), or 200 mM NaCl (root irrigation). Tissue samples were collected at 0, 3, 6, 12, and 24 h post-treatment, with three biological replicates for each time point. Each biological replicate consisted of tissue pooled from one individually grown plant. All collected plant materials were immediately frozen in liquid nitrogen and stored at −80 °C. The remaining plants were vernalized (temperature: 4 °C) for 21 days, and then returned to the greenhouse until the flowering stage. Subsequently, various tissues, including roots, stems, leaves, flowers, and siliques, were harvested, frozen in liquid nitrogen, and stored at −80 °C.

### 4.2. Phylogenetic Tree and Collinearity Analysis

The genomic DNA and protein sequences of *JAZ* family members from *A. thaliana* and cruciferous vegetables were retrieved from the TAIR (https://www.arabidopsis.org/) and BRAD (http://www.brassicadb.cn/) databases. Multiple sequence alignment of the full-length JAZ proteins was performed using MEGA11. A phylogenetic tree was constructed via the Neighbor-Joining (NJ) method, with 1000 bootstrap replicates to assess branch node support [31]. For synteny and duplication analysis, whole-genome protein sequences of the selected species were analyzed using TBtools v2.388 [32], which integrates the MCScanX algorithm [33]. Homologous gene pairs were first identified by BLASTP (E-value < 1 × 10^−5^). MCScanX was then employed to detect syntenic blocks within and across genomes. *JAZ* gene pairs located within these conserved collinear blocks were classified as segmental duplications. Tandem duplication events were identified based on genomic proximity. Genes from the same phylogenetic subgroup were considered tandemly duplicated if two or more were located within a 100-kb genomic region on the same chromosome, with no more than one non-*JAZ* gene intervening.

### 4.3. Gene Location, Structure, and Protein Motifs

The chromosomal locations of *BrJAZ* genes were obtained from the BRAD database and visualized using TBtools. The gene structures (intron/exon organization) were determined by submitting the CDS and corresponding genomic sequences of the *BrJAZ* genes to the online tool GSDS 2.0 (https://gsds.gao-lab.org/). Furthermore, the conserved motifs within the BrJAZ proteins were identified using the MEME Suite (https://meme-suite.org/meme/, accessed on 23 February 2025). The search parameters were set to a maximum of 10 motifs and “Zero or One Occurrence Per Sequence(zoops)” was selected. The motif sequences and motif logos are presented in Table S5 and Figure S2, respectively.

### 4.4. Total RNA Isolation and Quantitative Real-Time PCR

Total RNA was isolated from plant samples using the FreeZol Reagent kit (Vazyme, Nanjing, China). Subsequently, 1 µg of total RNA was reverse-transcribed into cDNA using the HiScript III RT SuperMix kit (Vazyme, Nanjing, China). The qPCR reactions were performed in a 15 µL volume, which consisted of 7.5 µL ChamQ Universal SYBR qPCR Master Mix (Vazyme), 0.5 µL of each forward and reverse primer, 3 µL of diluted cDNA template, and 3.5 µL of ddH_2_O. All reactions were run on the qTOWER2.0 system (Analytik Jena, Jena, Germany). The qPCR cycling consisted of an initial denaturation at 95 °C for 3 min, followed by 40 cycles of denaturation at 95 °C for 10 s and annealing/extension at 60 °C for 30 s. A melt curve analysis was subsequently carried out by heating from 65 °C to 95 °C, with continuous fluorescence measurement to verify amplification specificity. *BrACTIN* was used as the reference gene for normalization, and all primer sequences are listed in Supplementary Table S6.

### 4.5. Gene Clone and Vector Constructions

*BrJAZ1a* and *BrJAZ6b* were amplified from a Chinese cabbage inbred line cDNA using gene-specific primers (listed in Table S4). The resulting PCR products were then inserted into the plant expression vector pCambia1302 using the ClonExpress II One Step Cloning Kit (Vazyme Biotech Co., Ltd., Nanjing, China; Catalog No.: C112-02).

### 4.6. Plant Transformations

The recombinant plasmids were first introduced into *Agrobacterium tumefaciens* strain GV3101 via electroporation. These transformed agrobacteria were then used to infect *A. thaliana* plants via the floral dip method [34]. Transgenic seeds were surface-sterilized and sown on 1/2 MS medium containing 20 mg/L hygromycin for selection. After one week, resistant seedlings were transplanted into a 1:1 mixture of vermiculite and soil and grown in a controlled greenhouse under a 16-h light (22 °C)/8-h dark (19 °C) cycle. Putative transgenic plants were confirmed by both PCR and qPCR analyses using the primers listed in Tables S6 and S4. For stress treatments, T3 generation transgenic seedlings were grown to the five-leaf stage and then subjected to either high salt (200 mM NaCl, root irrigation) for 0, 3, 6, 12, and 24 h for enzymatic assays or to drought stress simulated by 15% PEG6000 for three weeks for phenotypic observation. During cultivation, seedlings were manually watered (root irrigation). All treatments at each time point were conducted with three biological replicates. For all expression and physiological assays, biological replicates refer to samples collected from independent plants grown under identical conditions. Quantitative real-time PCR (qPCR) and enzyme activity data were analyzed with GraphPad Prism 10 software and presented as mean ± standard deviation (SD) of at least three biological replicates. Statistical significance was determined by two-way analysis of variance (ANOVA) followed by appropriate multiple comparisons tests. Differences with *p* < 0.05 were considered statistically significant. For subcellular localization, the fusion constructs (*BrJAZ1a*-GFP and *BrJAZ6b*-GFP) were transiently expressed in tobacco leaves via agrobacterium-mediated infiltration. GFP signals were observed using a confocal microscope 72 h after infiltration.

### 4.7. Enzyme Activities

For the measurements of SOD and POD enzyme activities, 0.5 g of *A. thaliana* tissue from each treatment was harvested, flash-frozen in liquid nitrogen, and ground to a powder. The total protein content of each sample was quantified using a BCA Protein Assay Kit (Boxbio, Beijing, China) with bovine serum albumin (BSA) as the standard, according to the manufacturer’s protocol. The value was used to normalize the subsequent enzyme activity results, which were determined using specific commercial assay kits (Boxbio, Beijing, China) and are expressed as units per milligram of protein.

## Figures and Tables

**Table 1:** The information about the BrJAZ members.

Name	ID	Position	Genome (bp)	Exon	CDS (bp)	Protein (aa)	MW (Da)	PI
BrJAZ1a	BraA06g014970.3.5C	A06	2797	4	765	254	27,300	9.85
BrJAZ1b	BraA08g028950.3.5C	A08	2038	4	780	259	28,310	9.49
BrJAZ1c	BraA09g058470.3.5C	A09	1909	4	969	322	36,007	9.95
BrJAZ2a	BraA02g023330.3.5C	A02	1516	5	723	240	26,198	9.21
BrJAZ2b	BraA07g039730.3.5C	A07	1618	5	738	245	26,810	9.2
BrJAZ3a	BraA01g036030.3.5C	A01	3096	7	1071	356	37,877	9.37
BrJAZ3b	BraA05g030660.3.5C	A05	3273	7	1008	335	35,868	9.51
BrJAZ5a	BraA06g013220.3.5C	A06	1466	4	357	118	13,379	7.9
BrJAZ5b	BraA08g029870.3.5C	A08	1573	4	813	270	30,032	8.32
BrJAZ5c	BraA09g059410.3.5C	A09	1762	4	900	299	33,021	9.12
BrJAZ6a	BraA02g021780.3.5C	A02	1710	4	822	292	32,429	9.1
BrJAZ6b	BraA07g037970.3.5C	A07	1812	4	825	274	30,596	9.08
BrJAZ7a	BraA03g018070.3.5C	A03	342	1	342	113	13,014	8.74
BrJAZ7b	BraA04g025940.3.5C	A04	501	2	360	119	13,705	9.76
BrJAZ7c	BraA05g010440.3.5C	A05	556	2	351	116	13,144	9.64
BrJAZ8a	BraA08g023940.3.5C	A08	586	3	393	130	14,972	9.85
BrJAZ8b	BraA09g036440.3.5C	A09	582	3	402	133	15,329	9.62
BrJAZ9a	BraA07g030900.3.5C	A07	1518	5	639	212	23,526	9.03
BrJAZ9b	BraA07g036560.3.5C	A07	2677	7	804	267	28,813	9.76
BrJAZ10a	BraA02g004800.3.5C	A02	1532	4	555	187	21,005	10.07
BrJAZ10b	BraA03g005750.3.5C	A03	1783	4	552	183	20,569	9.95
BrJAZ10c	BraA10g025610.3.5C	A10	1557	5	591	196	21,819	9.91
BrJAZ12a	BraA02g009340.3.5C	A02	1810	5	666	221	23,083	4.97
BrJAZ12b	BraA10g019850.3.5C	A10	1431	4	528	195	20,395	7.93
BrJAZ13	BraA05g025740.3.5C	A05	549	3	372	123	14,005	9.9

## Data Availability

The datasets generated during and/or analyzed during the current study are not publicly available due Laboratory Requirements but are available from the corresponding author on reasonable request.

## Supplementary Materials

The following supporting information can be downloaded at: https://www.mdpi.com/article/10.3390/ijms27010289/s1.
