# Supplementary material for: Genome-Wide Analysis of the JAZ Family in Brassica rapa and the Roles of BrJAZ1a and 6b in Response to Stresses
Source: Int J Mol Sci. 2025 Dec 27;27(1):289. doi: 10.3390/ijms27010289 (PMC12786106; doi:10.3390/ijms27010289)
Supplement: Supplementary file 1 [file ijms-27-00289-s001.zip › ijms-4039982-supplementary.pdf]

Table S1 The homologous genes of *BrJAZ* in *A. thaliana*

Name of *B.rapa*	ID of *B.rapa*	ID of *A. thaliana*	Name of *A. thaliana*
BrJAZ1a	BraA06g014970.3.5C		
BrJAZ1b	BraA08g028950.3.5C		
BrJAZ1c	BraA09g058470.3.5C	AT1G19180	*JAZ1*
BrJAZ2a	BraA02g023330.3.5C		
BrJAZ2b	BraA07g039730.3.5C	AT1G74950	*JAZ2*
BrJAZ3a	BraA01g036030.3.5C		
BrJAZ3b	BraA05g030660.3.5C	AT3G17860	*JAZ3*
BrJAZ5a	BraA06g013220.3.5C		
BrJAZ5b	BraA08g029870.3.5C		
BrJAZ5c	BraA09g059410.3.5C	AT1G17380	*JAZ5*
BrJAZ6a	BraA02g021780.3.5C		
BrJAZ6b	BraA07g037970.3.5C	AT1G72450	*JAZ6*
BrJAZ7a	BraA03g018070.3.5C		
BrJAZ7b	BraA04g025940.3.5C		
BrJAZ7c	BraA05g010440.3.5C	AT2G34600	*JAZ7*
BrJAZ8a	BraA08g023940.3.5C		
BrJAZ8b	BraA09g036440.3.5C	AT1G30135	*JAZ8*
BrJAZ9a	BraA07g030900.3.5C		
BrJAZ9b	BraA07g036560.3.5C	AT1G70700	*JAZ9*
BrJAZ10a	BraA02g004800.3.5C		
BrJAZ10b	BraA03g005750.3.5C		
BrJAZ10c	BraA10g025610.3.5C	AT5G13220	*JAZ10*
BrJAZ12a	BraA02g009340.3.5C		
BrJAZ12b	BraA10g019850.3.5C	AT5G20900	*JAZ12*
BrJAZ13	BraA05g025740.3.5C	AT3G22275	*JAZ13*

Table S2 The collinear pair of *BrJAZ* members in *B.rapa*

B.rapa	*B.rapa*
BraA01g036030.3.5C	BraA05g030660.3.5C
BraA02g004800.3.5C	BraA03g005750.3.5C.2
	BraA10g025610.3.5C
BraA02g009340.3.5C	BraA10g019850.3.5C.2
BraA02g021780.3.5C	BraA06g013220.3.5C
	BraA07g037970.3.5C
	BraA08g029870.3.5C
	BraA09g059410.3.5C
BraA02g023330.3.5C	BraA06g014970.3.5C
	BraA07g039730.3.5C
	BraA07g029060.3.5C
	BraA08g028950.3.5C
	BraA09g058470.3.5C
BraA03g018070.3.5C	BraA04g025940.3.5C
	BraA05g010440.3.5C
BraA04g025940.3.5C	BraA05g010440.3.5C

	BraA08g023940.3.5C
	BraA09g036440.3.5C
	BraA08g023940.3.5C
BraA05g010440.3.5C	BraA09g036440.3.5C
	BraA07g037970.3.5C
BraA06g013220.3.5C	BraA08g029870.3.5C
	BraA09g059410.3.5C
	BraA08g028950.3.5C
BraA06g014970.3.5C	BraA09g058470.3.5C
BraA07g030900.3.5C	BraA07g036560.3.5C
	BraA08g029870.3.5C
	BraA09g059410.3.5C
BraA07g037970.3.5C	BraA08g028950.3.5C
	BraA09g058470.3.5C
BraA08g023940.3.5C	BraA09g036440.3.5C
BraA08g028950.3.5C	BraA09g058470.3.5C
BraA08g029870.3.5C	BraA09g059410.3.5C

Table S3 The collinear pair of *JAZ* members in *B.rapa*，*A.thaliana*，*B. napus*，and *B.oleracea*

B. rapa	*A.thaliana*	*B. rapa*	*B.oleracea*	*B. rapa*	*B. napus*
BraA02g021780.3.5C		BraA01g036030.3.5C	Bo1g119100		BnaC05g35610D
BraA06g013220.3.5C			Bo5g121480	BraA01g036030.3.5C	BnaA05g22360D
BraA07g037970.3.5C			Bo2g011150		BnaC01g34620D
BraA08g029870.3.5C		BraA02g004800.3.5C	Bo3g009050		BnaA01g27170D
BraA09g059410.3.5C	AT1G17380		Bo9g168900		BnaA10g20060D
BraA02g023330.3.5C		BraA02g009340.3.5C	Bo2g023250		BnaC03g71460D
BraA06g014970.3.5C			Bo9g150390	BraA02g004800.3.5C	BnaC09g43860D
BraA08g028950.3.5C			Bo2g076750		BnaC02g04570D
BraA09g058470.3.5C			Bo5g023780		BnaA02g01520D
BraA03g018070.3.5C		BraA02g021780.3.5C	Bo6g115920		BnaA03g04250D
BraA04g025940.3.5C	AT1G30135		Bo8g067140	BraA02g009340.3.5C	BnaA10g14660D
BraA05g010440.3.5C			Bo8g104890		BnaC09g36990D
BraA08g023940.3.5C		BraA02g023330.3.5C	Bo2g082410		BnaA02g05120D

BraA09g036440.3.5C			Bo5g027170		BnaC08g37780D
BraA01g036030.3.5C	AT1G48500		Bo6g119140		BnaA08g23150D
BraA05g030660.3.5C			Bo6g086720		BnaC06g33640D
BraA07g036560.3.5C	AT1G70700.3		Bo8g068340		BnaA06g11690D
BraA07g030900.3.5			Bo8g102890		BnaC05g13540D
BraA02g021780.3.5C			Bo3g027780		BnaA07g30200D
BraA06g013220.3.5C		BraA03g018070.3.5C	Bo4g182750		BnaA02g15990D
BraA07g037970.3.5C			Bo4g039500		BnaC08g18640D
BraA08g029870.3.5C			Bo3g027780		BnaC08g36840D
BraA09g059410.3.5C	AT1G72450		Bo3g147640		BnaA08g22180D
BraA02g023330.3.5C		BraA04g025940.3.5C	Bo4g182750	BraA02g023330.3.5C	BnaC06g35750D
BraA07g039730.3.5C			Bo4g039500		BnaC06g22690D
BraA08g028950.3.5C			Bo5g062450		BnaA06g13250D
BraA09g058470.3.5C		BraA05g010440.3.5C	Bo3g027780		BnaC05g14810D
BraA03g018070.3.5C	AT2G34600		Bo4g039500		BnaA07g31880D

BraA04g025940.3.5C	
BraA05g010440.3.5C	
BraA08g023940.3.5C	
BraA09g036440.3.5C	
BraA01g036030.3.5C	AT3G17860
BraA05g030660.3.5C	
BraA05g025740.3.5C	AT3G22275
BraA02g009340.3.5C	AT3G43440
BraA02g004800.3.5C	AT5G13220
BraA10g025610.3.5C	
BraA02g009340.3.5C	AT5G20900

	Bo4g182750		BnaA07g21950D
	Bo5g062450		BnaA02g17040D
BraA05g025740.3.5C	Bo3g080090		BnaCnng08230D
	Bo5g098140		BnaC04g10630D
BraA05g030660.3.5C	Bo1g119100		BnaA04g20270D
	Bo5g121480	BraA03g018070.3.5C	BnaA05g09200D
	Bo2g076750		BnaC03g19040D
	Bo5g023780		BnaA03g15910D
BraA06g013220.3.5C	Bo6g115920		BnaA08g17540D
	Bo8g067140		BnaA09g26390D
	Bo8g104890		BnaC04g10630D
	Bo2g082410	BraA04g025940.3.5C	BnaC05g23060D
BraA06g014970.3.5C	Bo5g027170		BnaA04g20270D
	Bo8g068340		BnaA05g09200D
	Bo8g102890		BnaC03g19040D
BraA07g030900.3.5C	Bo2g068460		BnaA03g15910D

	Bo6g094620		BnaA08g17540D
	Bo6g112300		BnaA09g26390D
	Bo2g068460		BnaC04g10630D
BraA07g036560.3.5C	Bo6g112300	BraA05g010440.3.5C	BnaC05g23060D
	Bo6g094620		BnaA04g20270D
	Bo2g076750		BnaA05g09200D
	Bo5g023780		BnaC03g19040D
BraA07g037970.3.5C	Bo6g115920		BnaA03g15910D
	Bo8g067140		BnaC05g30270D
	Bo8g104890	BraA05g025740.3.5C	BnaA05g17310D
	Bo2g082410		BnaC03g42730D
BraA07g039730.3.5C	Bo6g119140		BnaA03g36580D
	Bo6g086720		BnaC05g35610D
	Bo8g068340	BraA05g030660.3.5C	BnaA05g22360D
BraA08g023940.3.5C	Bo3g147640		BnaC01g34620D
	Bo4g039500		BnaA01g27170D

	Bo4g182750		BnaC08g37780D
	Bo5g062450		BnaA08g23150D
	Bo2g082410		BnaC06g33640D
	Bo5g027170	BraA06g013220.3.5C	BnaA06g11690D
	Bo6g086720		BnaC05g13540D
BraA08g028950.3.5C			
	Bo6g119140		BnaA07g30200D
	Bo8g102890		BnaA02g15990D
	Bo8g068340		BnaC08g18640D
	Bo2g076750		BnaC08g36840D
	Bo5g023780		BnaA08g22180D
BraA08g029870.3.5C	Bo6g115920		BnaC06g35750D
	Bo8g104890	BraA06g014970.3.5C	BnaA09g44300D
	Bo8g067140		BnaA06g13250D
	Bo3g147640		BnaC05g14810D
BraA09g036440.3.5C	Bo4g039500		BnaA07g31880D
	Bo4g182750		BnaA02g17040D

	Bo5g062450		BnaCnng08230D
	Bo2g082410		BnaC06g24560D
	Bo5g027170		BnaC06g31830D
	Bo6g086720		BnaA07g23750D
BraA09g058470.3.5C	Bo6g119140		BnaA07g28810D
	Bo8g102890		BnaC02g20120D
	Bo8g068340	BraA07g030900.3.5C	BnaC06g31830D
	Bo2g076750		BnaC06g24560D
	Bo5g023780		BnaA07g28810D
BraA09g059410.3.5C	Bo6g115920		BnaA07g23750D
	Bo8g104890		BnaC02g20120D
	Bo8g067140		BnaC08g37780D
	Bo2g011150		BnaA08g23150D
BraA10g025610.3.5C	Bo3g009050	BraA07g037970.3.5C	BnaC06g33640D
	Bo9g168900		BnaA06g11690D
			BnaC05g13540D

	BnaA07g30200D
	BnaA02g15990D
	BnaC08g18640D
	BnaA08g22180D
	BnaC06g35750D
	BnaC06g22690D
	BnaA07g31880D
	BnaA07g21950D
	BnaA02g17040D
	BnaCnng08230D
	BnaA08g17540D
	BnaA09g26390D
BraA08g023940.3.5C	BnaC04g10630D
	BnaC05g23060D
	BnaA04g20270D
	BnaA05g09200D

	BnaC03g59110D
	BnaC08g18640D
	BnaA08g22180D
	BnaC06g22690D
	BnaC06g35750D
BraA08g028950.3.5C	BnaA06g13250D
	BnaC05g14810D
	BnaA07g21950D
	BnaA07g31880D
	BnaA02g17040D
	BnaC08g37780D
	BnaA08g23150D
BraA08g029870.3.5C	BnaC06g33640D
	BnaA06g11690D
	BnaC05g13540D
	BnaA07g30200D

	BnaA02g15990D
	BnaA08g17540D
	BnaA09g26390D
BraA09g036440.3.5C	BnaC04g10630D
	BnaC05g23060D
	BnaA04g20270D
	BnaA05g09200D
	BnaC08g18640D
	BnaA08g22180D
	BnaC06g22690D
	BnaC06g35750D
BraA09g058470.3.5C	BnaA06g13250D
	BnaC05g14810D
	BnaA07g21950D
	BnaA07g31880D
	BnaA02g17040D

	BnaCnng08230D
	BnaC08g37780D
	BnaA08g23150D
	BnaC06g33640D
BraA09g059410.3.5C	BnaA06g11690D
	BnaC05g13540D
	BnaA07g30200D
	BnaA02g15990D
	BnaA10g20060D
	BnaC03g71460D
	BnaC09g43860D
BraA10g025610.3.5C	BnaC02g04570D
	BnaA02g01520D
	BnaA03g04250D

Table S4 Primers for Vector Construction

Gene No.	Forward (5'-3')	Reverse (5'-3')
JAZ1	ATGTCGAGTTCTATGGAGTGCTC	CATATCAGCTGCTAAACCAAGCC
JAZ6	ATGTCGACCGGACAAGCAC	AAGCTTGAGTTCGAGATCTTTTG
pCAMBIA1302-JAZ1	ctatgacatgattacgaattcATGTCGAGTTCTATGGAGTGCTCTG	tgcctgcaggtcgactctagaTCACATATCAGCTGCTAAACCAAGC
pCAMBIA1302-JAZ6	ctatgacatgattacgaattcATGTCGACCGGACAAGCACC	cctgcaggtcgactctagaCTAAAGCTTGAGTTCGAGATCTTTTG
pCAMBIA1300-cFLAG-JAZ1	cgcggtggcggccgctctagaATGTCGAGTTCTATGGAGTGCTCTG	gtcgacggtatcgataagcttTCACATATCAGCTGCTAAACCAAGC
pCAMBIA1300-cFLAG-JAZ6	5'-cgcggtggcggccgctctagaATGTCGACCGGACAAGCACC	gtcgacggtatcgataagcttCTAAAGCTTGAGTTCGAGATCTTTTG

Table S5 Motif sequences

Motif	Sequence
Motif1	VELPIARRASLHRFLEKRKDRVTSKAPYQ
Motif2	ESSTAQLTIFYGGKVCVFBDFPAEKAKEI
Motif3	EKPSFSRTCSRLSRYLKEKGSFGDJSLGMSCKPDVNL
Motif4	MLLAGNGSPMPQVFSPPQTHQQVVHHARASVDSSAMPPSFMPTVSYLSPE
Motif5	QFLSFRPSQDDRHRKPGNYHLPHSGSFMPSSVADVYDSNRR
Motif6	LQSHYPPGGKSFINNGINSQPFVGVPIMAPPISVLPPPGSIVGTTDIRSS
Motif7	MERDFLGLGSKQSPNTVKEET
Motif8	MQCNKDLELRL
Motif9	PYSSVQGARMFPSSYQHNZTIAVSMKRP
Motif10	LDRRRSFREIQGAISKIDPEIIKSLLASGANRSDSSTRSLSVPSTPKEDH

Table S6 Primers of qPCR

Name	Forward primer (5'-3')	Reverse primer (5'-3')
BrJAZ1a	TTCTCGCTGACGTGTAGTCG	GCATGACATCCCTAAGCTGAG
BrJAZ1b	TACTCGGAGATTCTCCAGGAA	AAGCTGCCGTTCTCTTTCAG
BrJAZ1c	AACGATGTCGAGTCCTATGGAG	CACGTCTGAGAGAAGCTCGG
BrJAZ2a	GTCTCGACAGCCAACGATGA	ACCTTATGCGTTGGTTTAACG
BrJAZ2b	AGCCGACGATGATGAATCTC	TTAATTGCCGAGGAATCACAT
BrJAZ3a	GTAGCTCAAATGGGTTCGGA	TGGATGCGTTAGTTTGGTTG
BrJAZ3b	GCCTCCTAGCTTCATGCCTA	AAGCCTCTTGCTCCGAGTAC
BrJAZ5a	ACGGAAAGACAGAGCAGTGG	TCTGGCTTGGGAGGATAATG
BrJAZ5b	AGAAATGAAGATGGTGAGGCA	CTTCAAGTAACGGCTGAGCA
BrJAZ5c	AGAAAATGCAAAGGCACTAGG	TTCAAGTAACGGCTGAGCAA
BrJAZ6a	TCATCGCAGTATGTCGAACG	TTCAAGTAGCGGCTGAACAA
BrJAZ6b	GACAAGCACCAGAGAAGTCCAA	GGCTGAGCAAACTACATCTCCG
BrJAZ7a	ATCCATCTCCAAGCAAAAGCTATAC	TTCCATATCCCTACTCGCTAGTGA
BrJAZ7b	TCCACAACCAAAGAAAGAATCACA	TGCCCGTTGTAGAAAATGGTTAAT
BrJAZ7c	GCAAGTGGAGACATGAAAGAAAAA	GCCGTCTGAACTTTCCAATGA
BrJAZ8a	TGTCCTTACAACTCAGAAGATGGAGA	AGACGAAGTTCCAAGTCGCAG
BrJAZ8b	ATGGAGAAGAACTGCGACTTGG	ACAAGAAGAAGTTGGAAAAAGACGAA
BrJAZ9a	CGTTGACGATGATCGTGTCG	CTAGCTGCTTTCGTGCTGGA
BrJAZ9b	TGTCGGAGAACGAGGACTGAG	GCCTTTCCCCATTGTTTAGCT
BrJAZ10a	CAGACCAATAACGCTCCAAA	AATCGCACCTTGCATTTCTC
BrJAZ10b	TCGTCGCAGTTTCCGAGATA	TCCGGATGGATCTTAGAAATCG
BrJAZ10c	TACTGGTGCTAACCCTTCCGA	GACGGAACCGATAGAGATCTGGT
BrJAZ12a	AAATCGCCGGTAACGGAAC	CAGCAACAGATCCGTCGATG
BrJAZ12b	GAAAAGCAAAGCGAAACTGGA	CATTCTCGCTGAACTCGTCG
BrJAZ13	TCACTTCAATCTTGTCGCCG	CGTGGCTAAAGATTGACTAACCTTG
BrTubulin1	TACGTTCCTCGTGCCGTTCT	GCTGTCCATCGTACCAGGCT

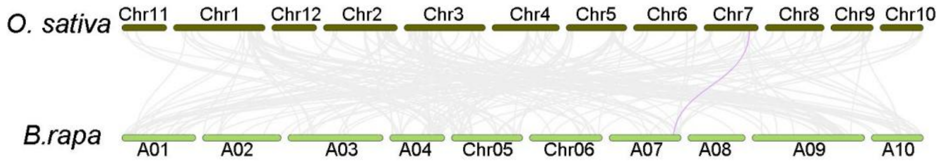

Figure S1 The synteny of JAZ members between *B. rapa* and *O. sativa.*

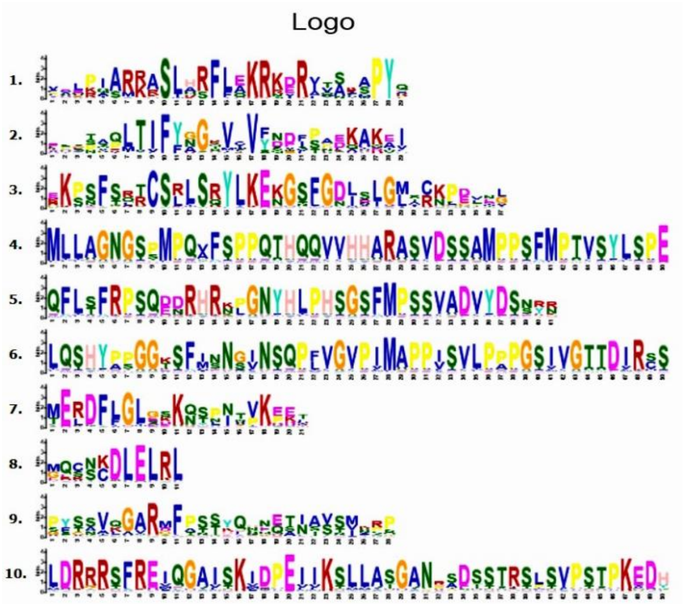

Figure S2 The logo of each motif
